# Formation and reinforcement of clusters composed of C_60 _molecules

**DOI:** 10.1186/1556-276X-6-80

**Published:** 2011-01-12

**Authors:** Shunji Kurosu, Takahiro Fukuda, Yuichi Shibuya, Toru Maekawa

**Affiliations:** 1 Bio-Nano Electronics Research Centre, Toyo University, 2100, Kujirai, Kawagoe, Saitama, 350-8585, Japan

## Abstract

We carry out two experiments: (1) the formation of clusters composed of C_60 _molecules via self-assembly and (2) the reinforcement of the clusters. Firstly, clusters such as fibres and helices composed of C_60 _molecules are produced via self-assembly in supercritical carbon dioxide. However, C_60 _molecules are so weakly bonded to each other in the clusters that the clusters are broken by the irradiation of electron beams during scanning electron microscope observation. Secondly, UV photons are irradiated inside a chamber in which air is filled at 1 atm and the above clusters are placed, and it was found that the clusters are reinforced; that is, they are not broken by electron beams any more. C_60 _molecules located at the surface of the clusters are oxidised, i.e. C_60_O*_n _*molecules, where *n *= 1, 2, 3 and 4, are produced according to time-of-flight mass spectroscopy. It is supposed that oxidised C_60 _molecules at the surface of the clusters may have an important role for the reinforcement, but the actual mechanism of the reinforcement of the clusters has not yet been clearly understood and therefore is an open question.

## Introduction

It is known that clusters composed of C_60 _molecules such as chains and sheets can be formed by polymerising C_60 _molecules via the irradiation of photons [1-13], application of high pressure and/or high temperature [3,5,6,14-17], or introduction of foreign atoms or molecules [18-20]. It is also known that C_60 _molecules can be modified with oxygen atoms and molecules [21-30].

The gas-liquid coexistence curves terminate at the critical points [31]. Incident light cannot penetrate fluids as they approach their critical points, known as critical opalescence, due to the formation of large molecular clusters [31]. It was recently shown that fibres, fibre networks, sheets and helices composed of C_60 _molecules were self-assembled by leaving C_60 _crystals in ethane, xenon or carbon dioxide under supercritical conditions for 24 h [32]. Those structures were formed via van der Waals interactions between C_60 _and the fluids' molecules.

In this letter, we create clusters composed of C_60 _molecules via self-assembly in supercritical carbon dioxide and reinforce the clusters by attaching oxygen atoms to the surface of C_60 _molecules.

## Experimental details

**Figure 1:**
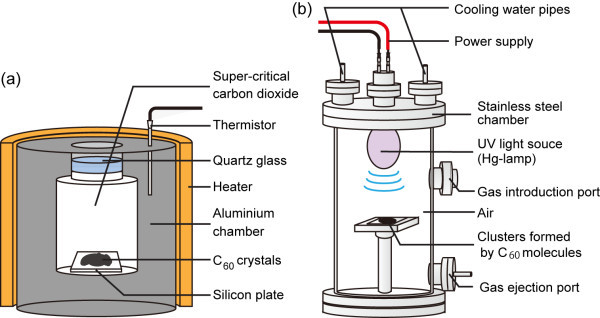
**Outline of the experimental apparatuses**. **(a) **Supercritical fluid chamber. Crystals composed of C_60 _molecules are placed on a silicon plate set at the bottom of the chamber. The chamber is then filled with carbon dioxide of critical density. The temperature is set at 36.0°C by a heater regulated by a PID controller. Crystals composed of C_60 _molecules are left in the chamber for 24 h. Clusters formed by C_60 _molecules are observed by an SEM. **(b) **UV light irradiation chamber. The clusters formed by C_60 _molecules are placed in the chamber which is filled with air at 1 atm. After irradiation of UV light for 3 h, the clusters are observed by an SEM and mass spectroscopic analysis is carried out.

Figure 1 shows an outline of the experimental apparatuses. We carried out two experiments. First, clusters composed of C_60 _molecules are produced by leaving C_60 _crystals in carbon dioxide under supercritical conditions for 24 h [32] (see Figure 1a). The inner volume of the supercritical fluid chamber made of aluminium was 11.7 ml. Of the crystals composed of C_60 _molecules, 0.2 mg was placed on the surface of a silicon plate set at the bottom of the supercritical fluid chamber and carbon dioxide of critical density was introduced into the chamber. The temperature of the fluid was set at 36.0°C by a heater installed around the chamber, which was regulated by a PID controller (C541, Technol Seven Co. Ltd., Tokyo, Japan). The temperature was monitored by a thermistor (SZL-64, Takara Thermistor Co. Ltd., Tokyo, Japan) embedded inside the chamber wall. Note that the critical temperature, pressure and density of carbon dioxide are respectively 31.0°C, 7.38 MPa and 468.0 kg m^-3 ^[33]. After the experiment, the fluid in the chamber was gradually released by controlling a valve switch. Clusters formed by C_60 _molecules were observed by a scanning electron microscope [SEM] (JSM-7400F, JEOL, Tokyo, Japan). Secondly, the clusters formed on the silicon plate were moved to another chamber made of stainless steel for irradiation of UV light (Figure 1b). The inner height and diameter of the chamber were 500 and 254 mm. The clusters were placed 150 mm under an Hg lamp (200 W, low-pressure Hg lamp, SEN Light Co. Ltd., Osaka, Japan). The chamber was filled with air at 1 atm and the air irradiated with UV light, the primary wavelengths of which were 184.9 and 253.7 nm, for 3 h. After the experiment, the chamber was vacuumed via the ejection port and fresh air was injected. The structures of the clusters were observed by an SEM. Mass spectroscopic analysis of the clusters was also carried out by matrix-assisted laser desorption ionisation time-of-flight mass spectroscopy (Brücker Daltonics, Autoflex, Bremen, Germany).

## Results

**Figure 2:**
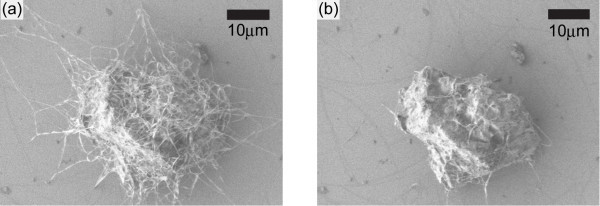
**Formation of clusters**. **(a) **Clusters formed by C_60 _molecules after having left the crystals composed of C_60 _molecules in carbon dioxide under supercritical conditions (36.0°C) for 24 h. **(b) **C_60 _molecules were so weakly bonded to each other that the clusters were broken by the electron beams, the energy flux of which was 1.5 nW nm^-2^, during the SEM observation.

First of all, clusters such as fibres and helices were formed by C_60 _molecules after having left the crystals composed of C_60 _molecules in carbon dioxide under supercritical conditions (36.0°C) for 24 h [32] (see Figure 2a). However, C_60 _molecules were so weakly bonded to each other that the clusters were broken by electron beams during the SEM observation (Figure 2b; see also Additional file 1 for the movie). Note that the accelerating voltage, current and diameter of the electron beams were 1 kV, 4.7 × 10^-2 ^nA and 2.0 nm, respectively, and therefore the energy flux of the electron beams was 1.5 nW nm^-2^.

**Figure 3:**
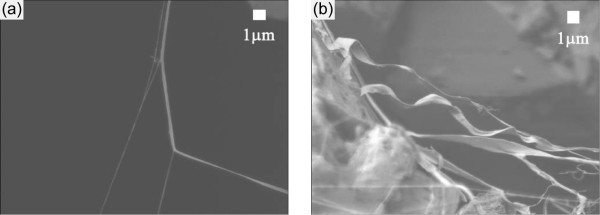
**Clusters after UV light irradiation for 3 h in a 1 atm air-filled chamber**. **(a) **Fibres. **(b) **Helices. Those structures were not broken by electron beams any more.

**Figure 4:**
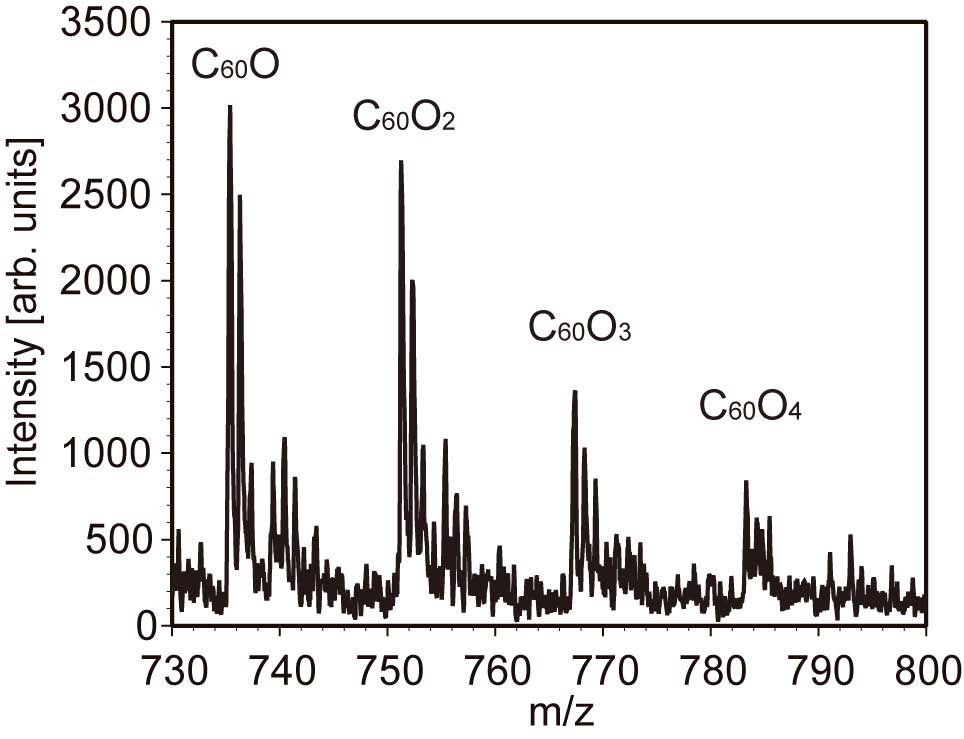
**Time-of-flight mass spectroscopy**. The clusters were composed of C_60 _molecules (not shown in the graph) and C_60_O*_n _*molecules (*n *= 1, 2, 3, 4). It is supposed that oxygen atoms are bonded to C_60 _molecules at the surface of the clusters.

**Figure 5:**
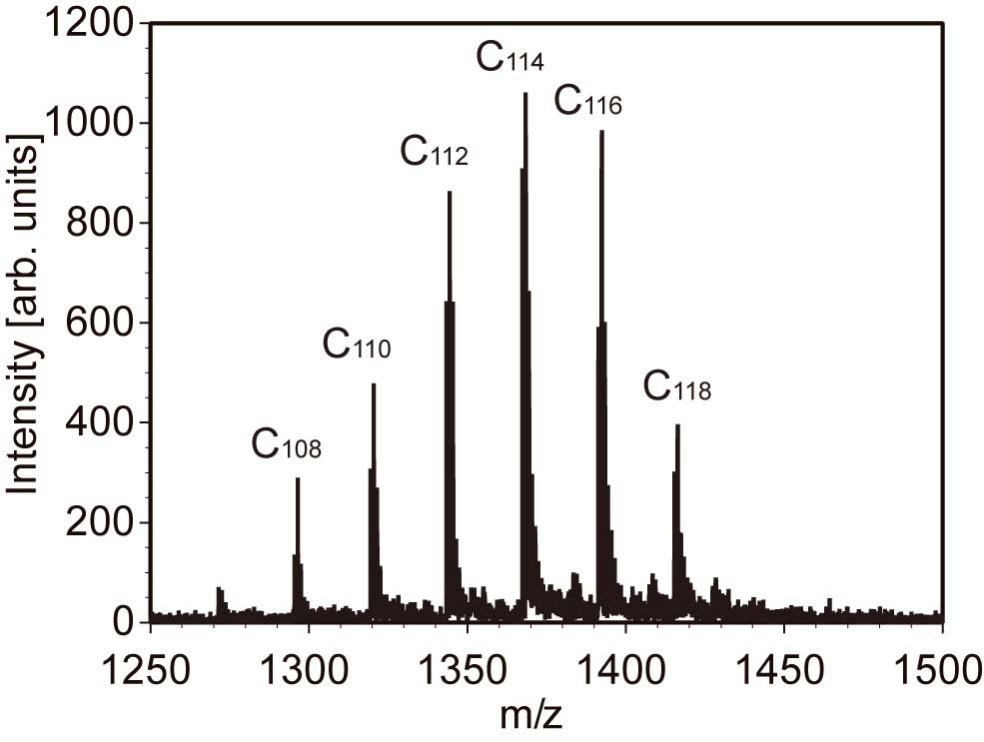
**Time-of-flight mass spectroscopy**. The fibres and helices were broken by electron beams and dimers were formed when the clusters were placed in a vacuumed chamber irradiated with UV light. The chamber was not filled with air.

**Figure 6:**
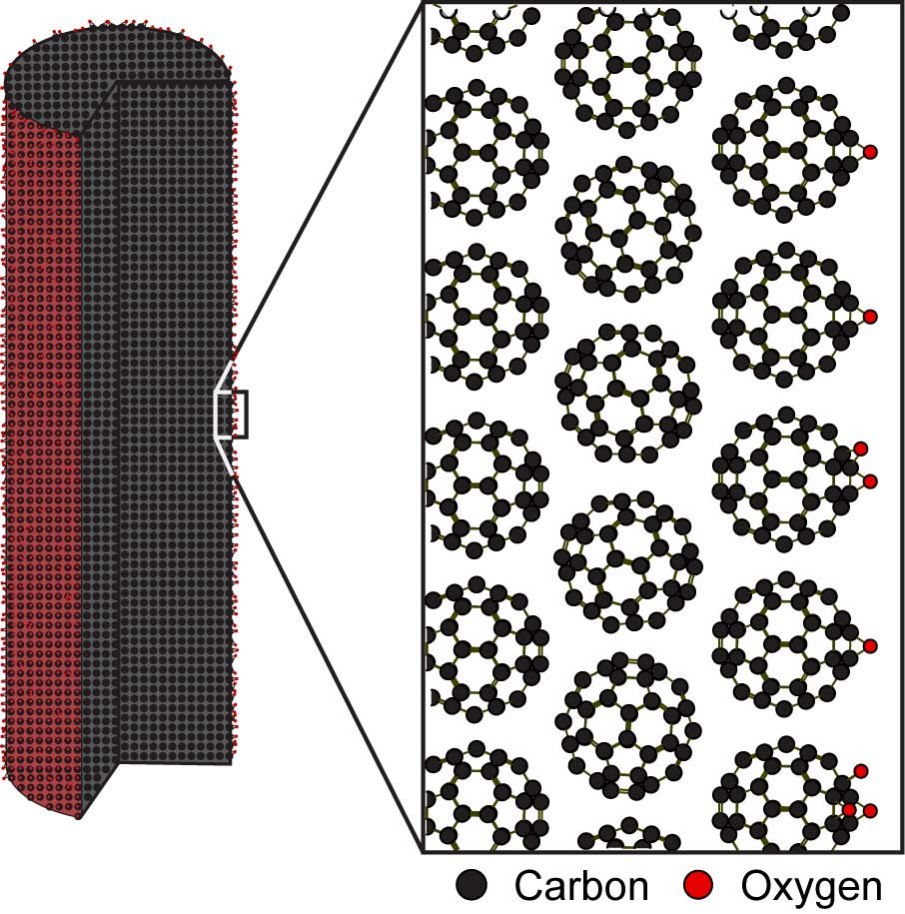
**Outline of a reinforced fibre composed of C_60 _molecules**. Originally, the fibre composed of C_60 _molecules was self-assembled in supercritical carbon dioxide (36.0°C). C_60 _molecules were so weakly bonded to each other that the fibre was broken by electron beams. The fibre was then placed in another chamber filled with air at 1 atm, which was irradiated with UV photons. C_60 _molecules located at the surface of the fibre were oxidised. The fibre was not broken any more by electron beams.

As mentioned, the clusters were placed in another chamber filled with air at 1 atm and irradiated with UV light in the chamber. Figure 3 shows the fibres and helices composed of C_60 _molecules after irradiation of UV light for 3 h. Those structures were not broken by electron beams anymore even when the accelerating voltage was raised up to 10 kV (see Additional file 1 for the movie). Note that those structures were broken by electron beams when the clusters were placed in a vacuumed chamber irradiated with UV light. Mass spectroscopic analysis of those clusters was carried out to investigate the component of the structures. Figure 4 shows the result of the mass spectroscopic analysis. Interestingly, C_60_O*_n _*molecules (where *n *= 1, 2, 3 and 4), that is, C_60 _molecules to which oxygen atoms are bonded, were detected, but neither C_120 _nor C_60_-O-C_60 _molecules were found. In other words, the reinforced clusters were not polymerised via a chemical bond. Note that when the chamber was vacuumed and UV light was irradiated, the clusters were broken as mentioned, but dimers such as C_108_, C_110_, C_112_, C_114_, C_116 _and C_118 _were created (see Figure 5). It is therefore supposed that air and irradiation of UV photons are essential for the reinforcement of clusters composed of C_60 _molecules. The dissociation energy of an oxygen molecule, O_2_→O + O, is 5.1 eV [34]; therefore, it is supposed that oxygen molecules in the chamber were dissociated by photons of 184.9-nm wavelength, the energy of which is 6.48 eV, and oxygen atoms were bonded to C_60 _molecules. The order of the diameter of the clusters being 10 nm, it is supposed that C_60 _molecules located at the surface of the clusters were oxidised (see Figure 6) and the clusters somehow reinforced. It is inferred that oxidised C_60 _molecules located at the surface of the clusters may have an important role for the reinforcement of the clusters.

We will be investigating the mechanism of the reinforcement of the clusters, that is, the role of oxidised C_60 _molecules (C_60_O*_n_*) located at the surface of the clusters, in the reinforcement process in detail, carrying out quantum mechanical calculations. We will also be measuring the electric, electronic, mechanical and thermal properties of the fibres and helices so that the clusters may be utilised for the development of nano electron devices, nano/microelectromechanical systems and micro-total analysis systems.

## Summary

We carried out two experiments: (1) Crystals composed of C_60 _molecules were placed in supercritical carbon dioxide (36.0°C), and it was found that fibres, fibre networks and helices composed of C_60 _molecules were self-assembled. Since C_60 _molecules in the clusters were bonded to each other via van der Waals interactions [32], the clusters were easily broken by the irradiation of electron beams during the SEM observation. (2) The clusters were placed in another chamber filled with air at 1 atm and irradiated with UV photons. Oxygen molecules were dissociated by UV photons, C_60 _molecules at the surface of the clusters were oxidised, and C_60_O*_n _*molecules were created. The clusters were not broken by the electron beams any more. It is supposed that C_60_O*_n _*molecules located at the surface of the clusters may have an important role in the reinforcement process, but the actual mechanism of the reinforcement of the clusters has not yet been clearly understood and therefore is an open question.

## Competing interests

The authors declare that they have no competing interests.

## Authors' contributions

SK designed the study and carried out the experiment. TF participated in the design of the study and performed SEM observation and mass spectroscopic analysis. YS participated in the reinforcement experiment. TM participated in the design of the study, coordinated the study and wrote the manuscript. All authors read and approved the final manuscript.

## Supplementary Material

Additional file 1 **Supplementary materials**. Supplementary Material 1 - SEM observation of clusters composed of C_60 _molecules which were self-assembled in supercritical carbon dioxide. The accelerating voltage of electron beams is 1.0 kV. The clusters are broken during the SEM observation. Supplementary Material 2 - SEM observation of clusters. C_60 _molecules located at the surface of the clusters were oxidised. The accelerating voltage of electron beams is 1.0 kV. The clusters are not broken any more during the SEM observation. Click here for file
